# Retinoic Acid Mediated Clearance of *Citrobacter rodentium* in Vitamin A Deficient Mice Requires CD11b+ and T Cells

**DOI:** 10.3389/fimmu.2018.03090

**Published:** 2019-01-08

**Authors:** Lindsay M. Snyder, Kaitlin L. McDaniel, Yuan Tian, Cheng-Hsin Wei, Mary J. Kennett, Andrew D. Patterson, A. Catharine Ross, Margherita T. Cantorna

**Affiliations:** ^1^Department of Veterinary and Biomedical Sciences, The Pennsylvania State University, University Park, PA, United States; ^2^CAS Key Laboratory of Magnetic Resonance in Biological Systems, State Key Laboratory of Magnetic Resonance and Atomic and Molecular Physics, National Centre for Magnetic Resonance in Wuhan, Wuhan Institute of Physics and Mathematics, University of Chinese Academy of Sciences, Wuhan, China; ^3^Department of Nutritional Sciences, The Pennsylvania State University, University Park, PA, United States

**Keywords:** vitamin A, macrophage, T cells, Citrobacter, retinoic acid

## Abstract

Vitamin A deficiency affects over 250 million preschool-age children worldwide and is associated with increased childhood mortality and risk of developing enteric infections. Vitamin A deficient (A–) mice developed chronic *Citrobacter rodentium* infection. A single oral dose of retinoic acid (RA) at d7 post-infection was sufficient to induce clearance of the pathogen in A– mice. RA treatment of A– mice induced *il17* expression in the colon. In A– mice, colonic IL-17 was primarily produced by CD11b+ cells; however, in A+ mice, the major source of colonic IL-17 was CD4+ T cells. To determine the cellular targets of vitamin A required for host resistance to *C. rodentium*, mice that express a dominant negative (dn) retinoic acid receptor (RAR) in T cells (T-dnRAR) or macrophage/neutrophils (LysM-dnRAR) were used. T-dnRAR mice had T cells that produced a robust intestinal IL-17 response and for 40% of the mice was enough to clear the infection. The remainder of the T-dnRAR mice developed a chronic infection. A– LysM-dnRAR mice developed early lethal infections with surviving mice becoming chronically infected. RA treatment of A– LysM-dnRAR mice was ineffective for inducing colonic IL-17 or clearing *C. rodentium*. Retinoid signaling is required in T cells and CD11b+ cells for complete elimination of enteric pathogens.

## Introduction

Vitamin A deficiency (A–) is an important public health problem in resource limited countries including areas in sub-Saharan Africa and southeastern Asia. The most recent World Health Organization studies estimate that 250 million pre-school age children worldwide are vitamin A deficient and these children have increased rates of enteric infection (1, 2). Vitamin A supplementation programs have been shown to effectively raise host vitamin A status and reduce the incidence and severity of enteric infections (3). Vitamin A deficiency is associated with severe enteric infections in both humans and mice (4). A– mice infected with *Citrobacter rodentium*, a gram negative murine enteric pathogen, developed a severe infection, including premature mortality of 40% of the A– mice (4). Surviving A– mice failed to clear the infection and became chronic carriers of *C. rodentium* (4). *C. rodentium* in mice results in a disease that models human enteropathogenic *Esherichia coli* infection and acute Th1/Th17-driven intestinal inflammation (5). Vitamin A sufficient (A+) mice cleared *C. rodentium* within 4 weeks of infection. Protection from early infection is provided by innate lymphoid cells (ILC) that produce IL-22 and IL-17 (ILC3) and clearance of infection requires robust Th17 cell responses (6, 7). The colons of IL17 knockout (KO) mice had exacerbated pathology at peak infection and higher bacterial burdens at d7, d14, and d21 post-infection (8). IL-17 expression levels in the colon were highest during peak and late infection, which corresponded with clearance of *C. rodentium* in WT mice (9). Host resistance to *C*. *rodentium* requires early IL-22 responses and late IL-17 production from T cells, which is necessary for clearance.

Vitamin A is a well described regulator of immune cells. Retinoic acid (RA) has been reported to suppress IFN-γ and IL-17 production from T cells *in vitro* (10, 11). The inhibition of IL-17 production by RA resulted in the induction of FoxP3 and IL-10 secreting regulatory T cells (10). RA induces homing of T and B cells to the gut by up-regulation of α4β7 and CCR9 expression (12, 13). RA reduced colonic inflammation caused by dextran sodium sulfate or *C. rodentium* infection and the reduced colonic inflammation corresponded with lower IL-17 expression (14–16). Paradoxically, A– mice infected with the intracellular parasite *Toxoplasma gondii* had lower IL-17 responses and RA treatments in this model induced more T cells that produced IL-17 (17). There are contradictory results on the effects of vitamin A status and RA treatments on IL-17 (15, 17).

We hypothesized that the effects of RA depended on the vitamin A status of the host. Here, the mechanisms and targets underlying RA-mediated clearance of *C. rodentium* in the A–host were determined. A single dose of RA was sufficient to promote host survival and clearance of *C. rodentium* infection in A– mice. Although RA was protective, retinoid repletion of the A– host resulted in an immunologically and metabolically distinct phenotype, different from both A+ and A– mice. RA mediated protection from *C. rodentium* was associated with an increase in colonic IL-17 production. Sixty percent of mice that expressed a dominant negative (dn) retinoic acid receptor (RAR) in T cells (T-dnRAR), developed a chronic *C. rodentium* infection. Infected A+ T-dnRAR mice had robust IL-17 production by T cells in the colon that for some mice (forty percent), was effective for inducing clearance of the infection. RA treatments of the T-dnRAR was ineffective for clearing *C. rodentium*. IL-17 production in the A– host was derived primarily from CD11b+ cells rather than T cells. RA treatment of A– LysM-dnRAR mice was ineffective for induction of CD11b+ mediated IL-17 or for clearance of the infection. The effects of retinoids in A– mice are distinct from those in A+ mice, and retinoid regulation of CD11b+ and T cell function contributes to effective protection of the host from *C. rodentium* infection.

## Methods

### Animals

C57BL/6J WT and Lck-Cre and LysM-Cre mice were obtained from Jackson Laboratories (Bar Harbor, ME). The dnRAR fl/fl mice (18) were a gift from Randolph J. Noelle (Dartmouth Medical School, Lebanon, NH). The dnRAR blocks retinoid signaling through all 3 RAR isoforms (19). dnRAR fl/fl cre^−/−^ (WT littermates) and dnRAR fl/fl cre +/– (T-dnRAR or LysM-dnRAR) were used for experiments. All mice were bred at the Pennsylvania State University (University Park, PA) according to university and IACUC guidelines. A+ and A– mice were generated by breeding C57BL/6J WT animals on lab prepared purified diets as described previously with and without retinyl acetate (25 μg/d) (4). A– mice were dosed with 37.5 μg of RA in small amounts of corn oil (10 μl) orally once at d7 post-infection (RA 1x), twice at d7 and d9 post-infection (RA 2x), or three times at d7, d9, and d11 post-infection (RA 3x). For some experiments A– mice were treated with RA three times weekly for 4 wk (metabolomics) or 8 wk (immune cell reconstitution). Serum retinol was quantified to verify the vitamin A status of experimental animals Supplementary Figure 1.

### UPLC Serum Retinol Quantification

Serum samples were saponified and quantified for total retinol concentration by ultra-performance liquid chromatography (UPLC) (20). Briefly, serum aliquots (30–100 μl) were incubated for 1 h in ethanol before adding 5% potassium hydroxide and 1% pyrogallol. After saponification in a 55°C water bath, hexanes (containing 0.1% butylated hydroxytoluene, BHT) and deionized water were added to each sample for phase separation. The total lipid extract was transferred and mixed with a known amount of internal standard, trimethylmethoxyphenyl-retinol (TMMP-retinol). Samples were dried under nitrogen and reconstituted in methanol for UPLC analysis. The serum total retinol concentrations were calculated based on the ratio of area under the curve between internal standard (known amount) and sample total retinol level (unknown).

### *C. rodentium* Infection

*C. rodentium* strain ICC169 (nalidixic acid resistant) was a kind gift from Gad Frankel (London School of Medicine and Dentistry, London, UK). Bacteria were grown and cultured in Difco Luria-Bertani broth and agar (LB; Becton, Dickinson, & Co., Franklin Lakes, NJ) containing 50 μg/mL nalidixic acid (Sigma Aldrich, St. Louis, MO, USA). Overnight cultures containing log phase bacteria were used to prepare inoculums. Individually housed mice were fasted overnight and then orally gavaged with 5 × 10^9^ CFU *C. rodentium* in 100 μl of sterile saline. Feces and organs were collected, homogenized, and plated in serial dilutions on LB agar plates containing nalidixic acid to quantify bacterial burdens.

### Flow Cytometry

Single cell suspensions of spleen, MLN, thymus, small intestinal (SI) intraepithelial lymphocytes (IEL) and colonic lamina propria (LP) cells were isolated as described previously (21–24). Peyer's patches were removed from SI, and both SI and colon tissues were cut longitudinally. Tissues were incubated twice (20 min at 37 C) with 1 mM 1,4 dithiothreitol (DTT, Sigma Aldrich) and 10 mM EDTA to release IEL cells. To obtain colon LP, the tissue was further minced and incubated for 1 h in collagenase (300 U/mL). IEL and LP cells were collected from the 40/80% interface of Percoll (Sigma Aldrich) gradients. One to two million cells were utilized for flow staining. Intracellular staining for IL-17 was done by stimulating 10^6^ colonic lymphocytes for 4 h with phorbol 12-myristate 13-acetate (50 ng/mL, Sigma, St. Louis, MO), and ionomycin (6 μg/mL) in the presence of brefeldin A (10 μg/mL, Sigma) St. Louis, MO). Cells were stained with fluorescein isothiocyanate (FITC) CD8β, FITC GL3 (γδ TCR), phycoerythrin PE GL3, PE-CF594 CD4, PE-CF594 GR-1, PE-Cy 5 T cell receptor β (TCRβ) (BD Biosciences, San Jose, CA), PE-Cy5 CD11b (eBioscience, San Diego, CA), PE-Cy7 F4/80, PE- PE-Cy7 CCR9, or PE-Cy7 CD8α (BioLegend, San Diego, CA). Intracellular cytokine staining was performed using PE IL-17 and the Foxp3/Transcription Factor Staining Buffer Set (eBioscience). Single positive and fluorescence minus one (FMO) controls were used for gating purposes. Cells were analyzed on an FC500 benchtop cytometer (Beckman Coulter, Brea, CA), and data was analyzed using FlowJo 7.6.5 software (Tree Star, Ashland, OR).

### Real-Time PCR

Whole tissues were snap frozen in liquid nitrogen and stored until processing. RNA was isolated using TriZOL (Invitrogen, Carlsbad, CA) as described in the manufacturer's protocol. Complementary DNA (cDNA) was created by reverse transcribing 4–5 μg of RNA using the TaqMan reverse transcription reagents kit (Applied Biosystems, Carlsbad, CA). RT-PCR was performed using SYBR green mix (BioRad, Hercules, CA) and the MyiQ Single-Color Real Time PCR machine (BioRad). Standards were prepared by serially diluting DNA products for each gene of interest. Gene expression levels were then normalized to one or more housekeeping genes (HPRT and/or GAPDH). Fold change values were reported relative to untreated or un-infected control tissues which were set at 1. Primers for RT-PCR are listed Supplementary Table 1.

### NMR

The liver samples for NMR was prepared as previously described (25). ^1^H NMR spectra were recorded at 298 K on a Bruker Avance III 600 MHz spectrometer equipped with an inverse cryogenic probe (Bruker Biospin, Germany). NMR spectra of liver samples were acquired for each employing the first increment of NOESY pulse sequence (NOESYPR1D) with the recycle delay (RD) of 2 s and mixing time (t_m_) of 100 ms. NMR spectra of samples were acquired using the Carr-Purcell-Meiboom-Gill sequence [RD-90°-(τ-180°-τ) n-acquisition]. The 90° pulse length was adjusted to about 10 μs for each sample and 64 transients were collected into 32 k data points for each spectrum with spectral width of 20 ppm. The chemical shift of ^1^H NMR spectra were referenced to TSP. Each bucketed region (0.004 ppm) was then normalized to the total sum of the spectral integrals prior to statistical data analysis. Principal component analysis (PCA) and orthogonal projection to latent structure-discriminant analysis (OPLS-DA) were carried out using the SIMCA-P+ software (Version 13.0, Umetrics, Sweden).

### Statistical Analyses

Statistical analyses were performed using GraphPad Prism (GraphPad, La Jolla, CA). Two-tailed student's *t*-tests were used to compare normally distributed data with only two groups. A student's *t-*test with Welch's correction was also used to compare data sets with unequal variances. Two-way ANOVA with Bonferroni's *post-hoc* test was used to compare fecal shedding curves and mRNA abundance through time and by treatment. A *P* ≤ 0.05 was used as the cut off for a significant change.

## Results

### A Single RA Dose Enables Clearance of *C. rodentium* Infection in A– Mice

**Figure 1:**
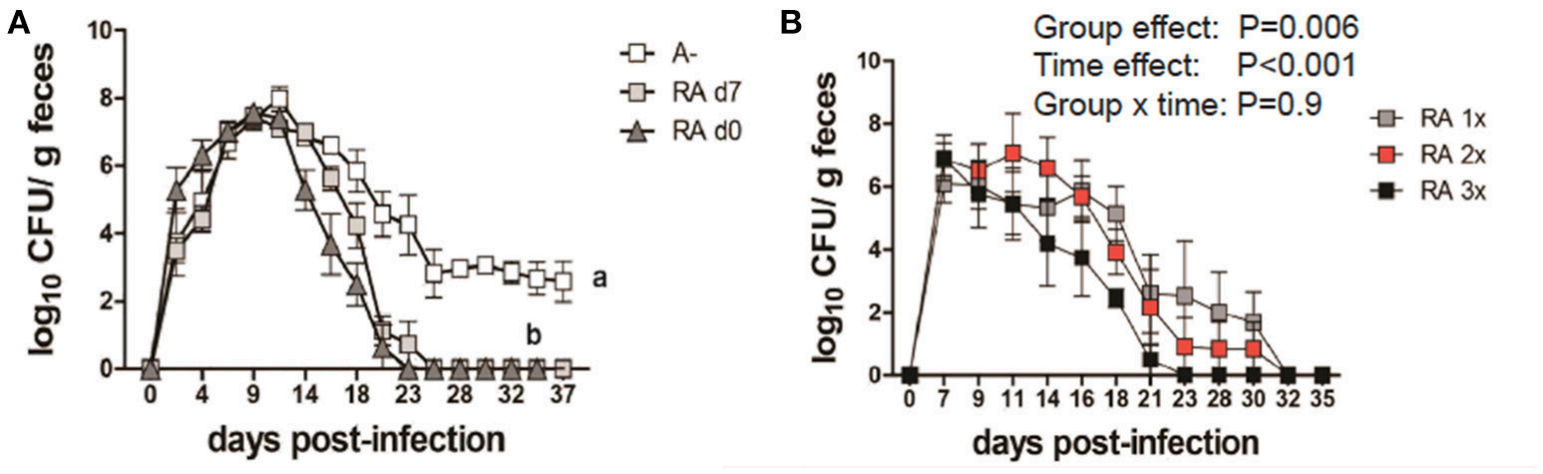
RA treatment enables clearance of *C. rodentium* infection in A– mice. A– WT mice were infected with *C. rodentium* and treated continuously with RA beginning at d0 or d7 **(A)** post-infection. **(B)** At d7 post-infection A– mice were dosed with RA once (RA 1x), twice (RA 2x), or three times (RA 3x). Values are the means ± SEM of *n* = 3–4 mice / group. Two-way ANOVA with Bonferroni *post-hoc* test. Groups with different letters are significantly different from each other, *P* < 0.01.

A– mice were infected with *C. rodentium* and left untreated (A–) or dosed with RA starting on the day of infection (RA d0) or at d7 post-infection (RA d7) and the RA dosing was continued throughout the experiment. RA dosing beginning at either d0 or d7 induced clearance of the *C. rodentium* infection (Figure 1A) with the kinetics of clearance similar to those in A+ WT mice (4). The untreated A– mice continued to shed *C. rodentium* out to d37 post-infection as reported previously (4). A– mice were treated with RA orally every 48 h once (RA 1x), twice (RA 2x), or three times (RA 3x) beginning on d7 post-infection (Figure 1B). RA 3x mice cleared the infection by d25 post-infection, while the RA 2x and RA 1x treatments took 30–32d to clear the *C. rodentium* infection (Figure 1B). RA 3x was more effective than RA 2x or RA 1x for the clearance of *C. rodentium*. As little as one dose of RA given to A– mice on d7 post-infection, resulted in clearance of *C. rodentium*.

### Distinct Metabolic and Immunologic Responses in A+, A–, and A– + RA Mice

**Figure 2:**
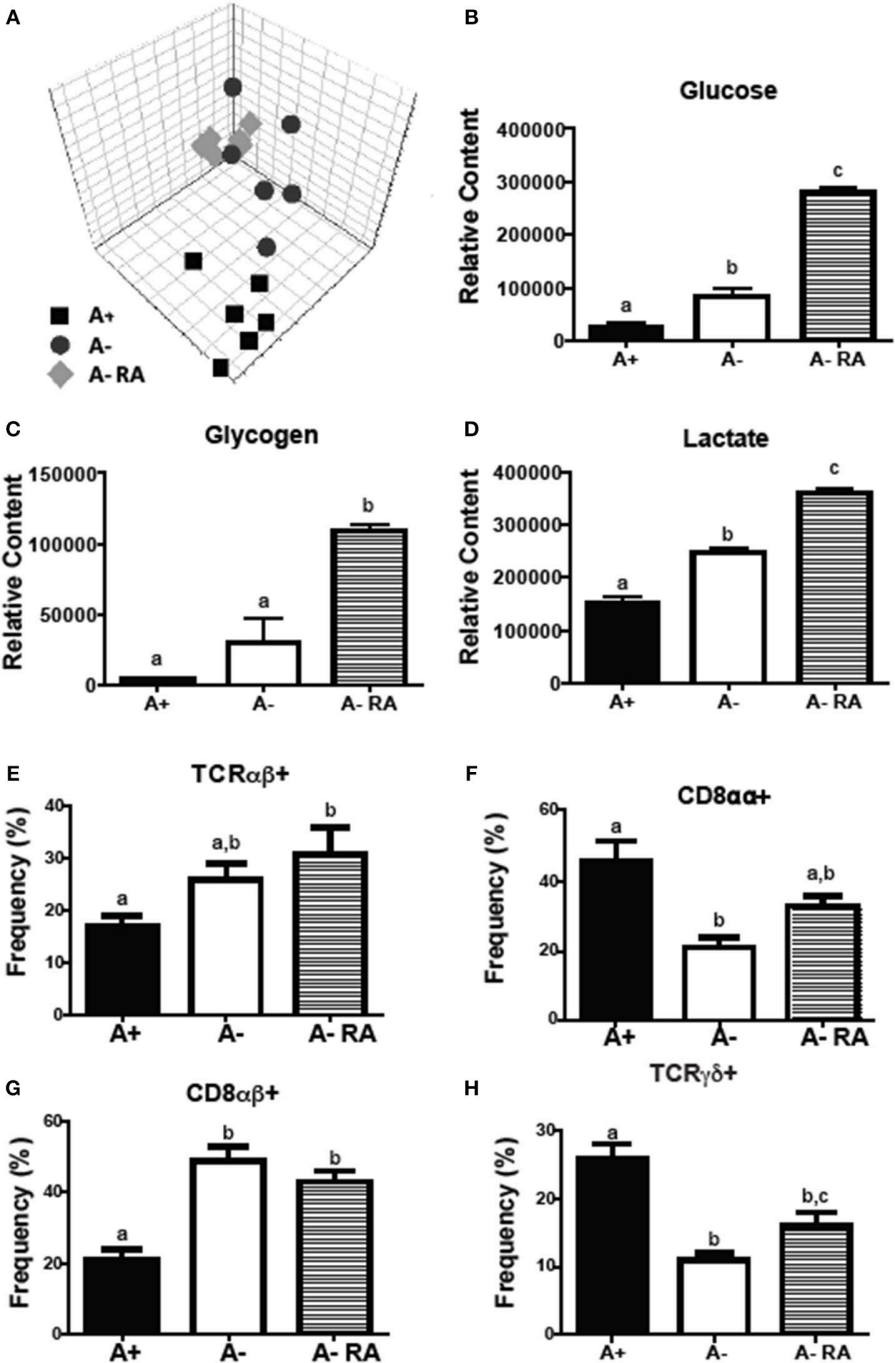
A+, A–, and A– RA mice have phenotypically distinct metabolite profiles and mucosal immune populations. **(A)** Liver PCA plot was generated using NMR data from A+, A–, and A– RA (4 weeks of RA dosing) mice (*n* = 6). Liver **(B)** glucose, **(C)** glycogen, and **(D)** lactate levels were quantified using ^1^H NMR (*n* = 6). Naïve A+, A–, and A– RA mice (8 weeks of RA dosing) were euthanized at 8–10 weeks of age to characterize SI IEL T cells. **(E)** TCRαβ+, **(F)** CD8αα+, **(G)** CD8αβ+, and **(H)** TCRγδ+ T cell frequencies. In the SI IEL (*n* = 8–9). Values are the means ± SEM of two to three independent experiments. One-way ANOVA with Bonferroni post-test, Groups with different letters are significantly different from each other, *P* < 0.05.

Livers were collected from A+, A– and A– RA mice for NMR-based metabolomics studies. Principle component analysis (PCA) revealed that A+ and A– mice had unique liver (Figure 2A) metabolic profiles that clustered separately from one another. RA dosed A– mice did not cluster with A+ or A– mice (Figure 2A). Instead, RA dosing created a third group with a unique liver metabolic profile (Figures 2B–D). Liver metabolites including several amino acids, glucose, glycogen, and lactate were higher in A– mice compared to A+ mice (Figure 2 and Supplementary Figure 2) (26). Branched chain amino acids, tyrosine, phenylalanine and histidine levels were reduced to A+ levels with the RA treatment of A– mice (Supplementary Figure 2). RA treatment of A– mice resulted in significantly higher liver glucose, glycogen, and lactate compared to A+ and A– mice (Figures 2B–D). RA treatment of A– mice increased glucose, glycogen and lactate levels in the liver over the already high levels in A– mice (Figures 2B–D). A– mice had fewer total cells in the SI than A+ and A– RA mice (Supplementary Figure 2E). A+ mice had fewer CD8αβ+ cells and more CD8αα+ and T cell receptor (TCR)γδ+ T cells than A– mice in the SI (Figures 2F–H). A– RA treated mice had significantly more TCRαβ+ cells and CD8αβ+ cells and significantly fewer TCRγδ+ T cells than A+ mice in the SI (Figures 2E–H). The frequencies of γδ+ T cells in the A– RA treated mice were significantly different compared to both the A+ and the A– mice (Figure 2H). A+, A–, and A– RA treated mice were three metabolically and immunologically distinct groups.

### RA 2x Treatment Induced *il17* in the Colons of A– Mice

**Figure 3:**
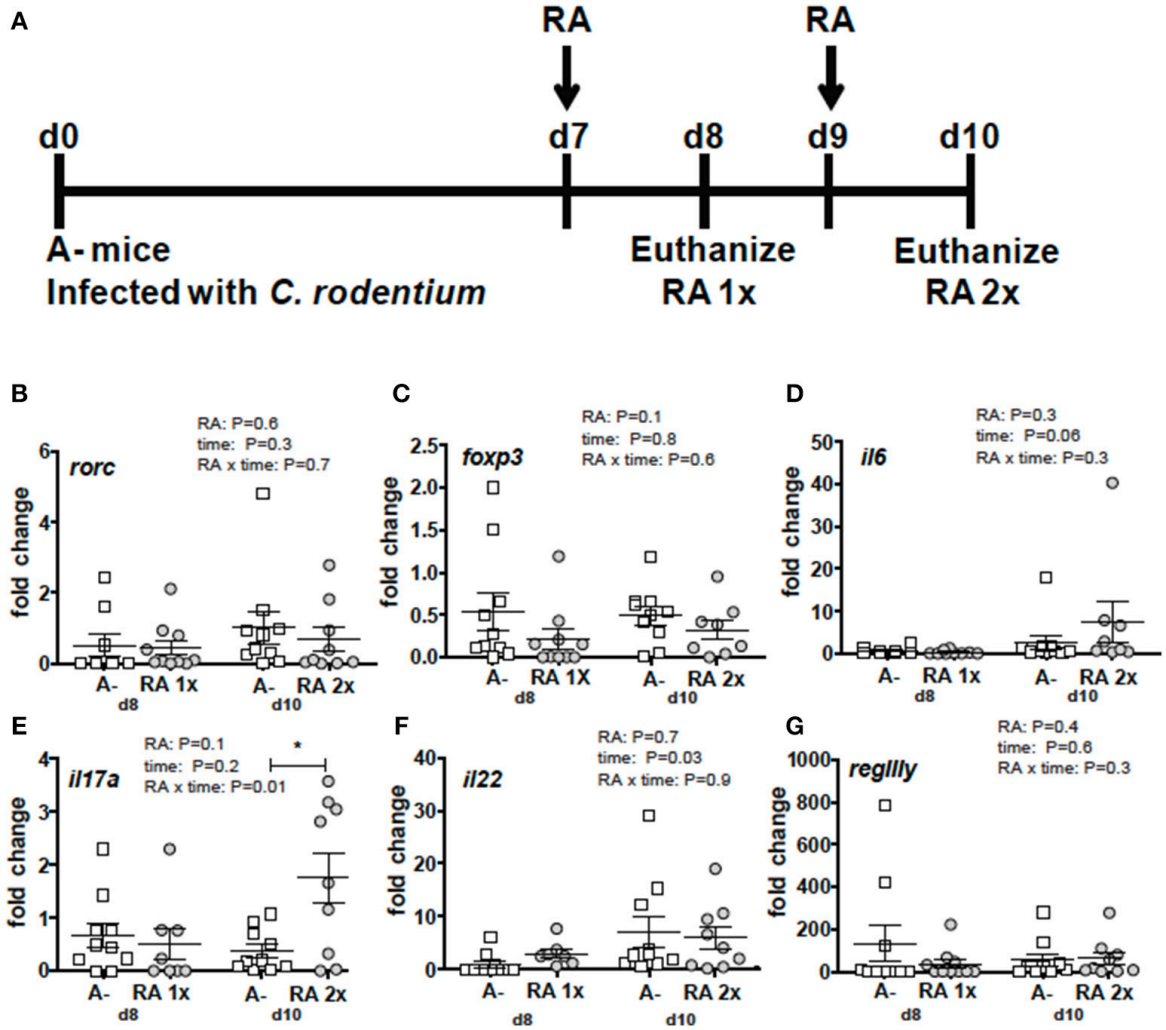
RA mediated induction of *il17* mRNA in the colon of A– mice. **(A)** Experimental design: *C. rodentium* infected A– mice were dosed with RA once at d7 (RA 1x) or twice at d7 and d9 post-infection (RA 2x). Mice were sacrificed on d8 (RA 1x) and d10 (RA 2x). **(B)** *rorc*, **(C)** *foxp3*, **(D)** *il6*, **(E)** *il17*, **(F)** *il22*, and **(G)** *regIII*γ expression in the colon following RA 1x or RA 2x. Values are mean ± SEM of two combined experiments and *n* = 8–10/group. Two-way ANOVA with Bonferroni *post-hoc* tests. ^*^P = 0.05.

**Figure 4:**
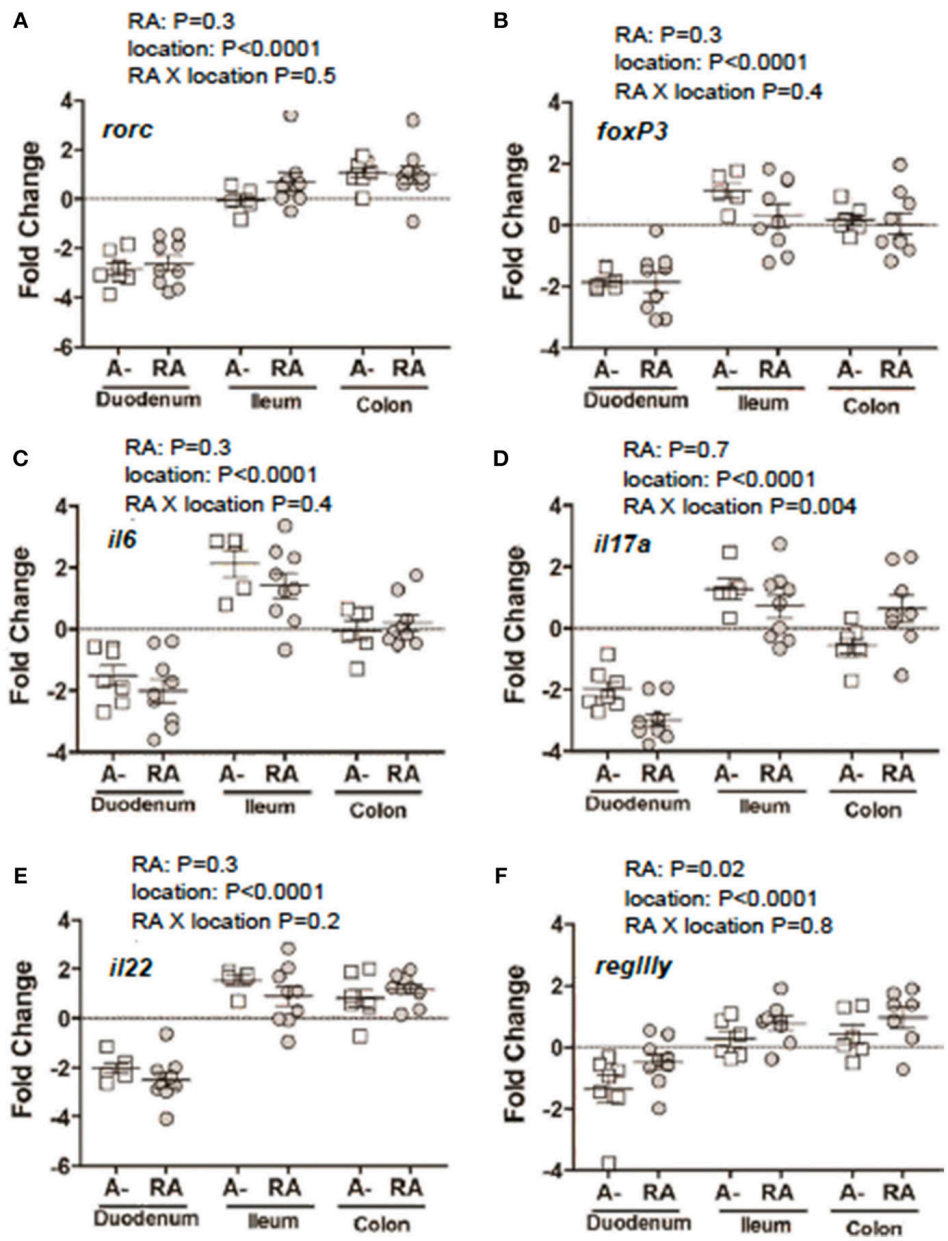
mRNA expression in the gastrointestinal tract of A– mice treated with or without RA. A– mice were infected, treated or not with RA 2x and sacrificed at d10-post-infection as shown in Figure 3A. **(A)** r*orc*, **(B)** *foxp3*, **(C)** *il6*, **(D)** i*l17*, **(F)** i*l22*, and **(E)** *regIII*γ expression in RA 2x treated A– mouse duodenum, ileum, and colon. Values were log transformed and are reported as means ± SEM from two independent experiments with *n* = 5–9 mice/group. Two-way ANOVA with Bonferroni *post-hoc* tests.

*C. rodentium* infected A– mice were untreated or dosed with RA 1x (d7) or RA 2x (d7 and d9) and sacrificed on d8 or d10 post-infection (Figure 3A). There was no effect of RA 1x or RA 2x on expression of *rorc, foxp3, il6, il22*, and *regIII* mRNA (Figures 3B–D,F,G). There was no effect of one dose of RA on colonic *il17* abundance (Figure 3E). RA 2x treatment significantly increased *il17* expression in the d10 infected colon (Figure 3E). To determine the effect of RA across the intestine, mRNA expression was measured in the duodenum, ileum, and colon of A– mice with and without RA 2x at d10 post-infection (Figure 4). The expression of *rorc, foxp3, il6, il17, il22*, and *regIII* mRNA was significantly lower in the duodenum than the ileum and colon of the *C. rodentium* infected mice (Figures 4A–F). There was no effect of the RA treatment on *rorc, foxp3, il6, or il22* mRNA expression (Figures 4A–C,E). RA treatment significantly enhanced *regIIIy* expression across the gastrointestinal tract (Figure 4F). RA treatment also enhanced *il17* expression in the colon but not the duodenum or ileum (Figure 4D). RA 2x treatment induced *il17* in the colon and *regIII* expression across the SI and colon of infected A– mice.

### Mice With T Cells That Have Blocked Retinoid Signaling Fail to Clear *C. rodentium*

**Figure 5:**
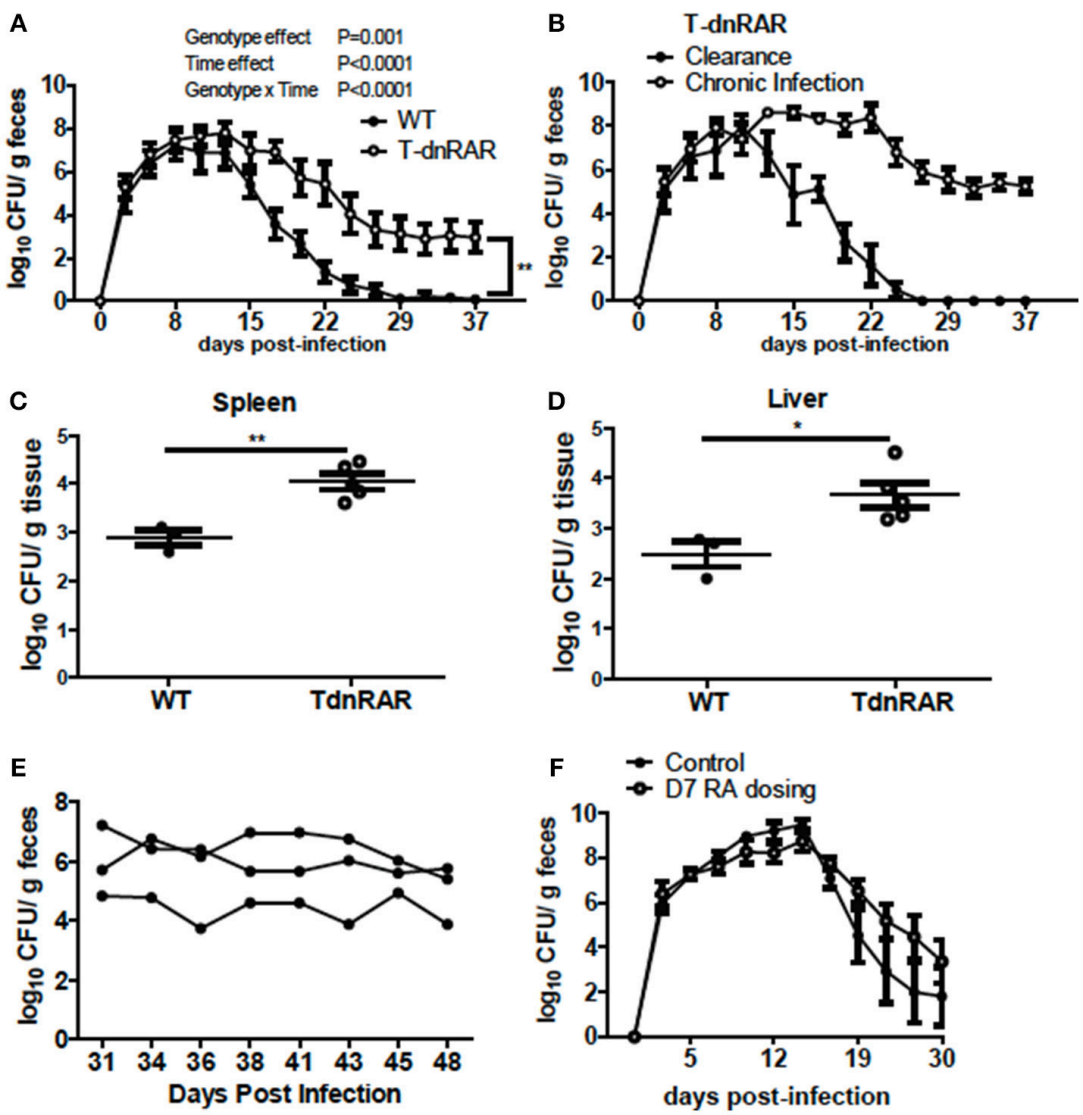
T-dnRAR mice fail to clear *C. rodentium* infection. **(A)** Fecal shedding kinetics of T-dnRAR and WT littermates infected with *C. rodentium* (*n* = 15–16 per group). **(B)** Fecal shedding in T-dnRAR that either cleared the infection (*n* = 6), or developed chronic infection (*n* = 9). **(C,D)** Spleen and liver CFU at d10 post-infection (*n* = 3–5). **(E)** Chronically infected T-dnRAR mice were treated with RA beginning at d31 post-infection. **(F)** Fecal shedding of untreated T-dnRAR mice and T-dnRAR mice that were RA dosed starting at d7 post-infection (*n* = 6–9). Values are the means ± SEM of two to three independent experiments. Two-way ANOVA **(A,B,E,F)** or Mann Whitney test **(C,D)** ^*^P < 0.05, ^**^P < 0.01.

To determine the role of retinoids in T cells for the clearance of *C. rodentium*, mice were generated in which retinoid signaling was blocked in T cells (T-dnRAR). The shedding kinetics of *C. rodentium* in the WT littermates (Figure 5A) matched that of A+ WT mice (4). Forty percent of T-dnRAR mice cleared the *C. rodentium* infection with the same kinetics as their WT littermates (Figure 5B). The other 60% of the T-dnRAR mice developed a very high and persistent infection that was not cleared by d37 (Figure 5B). There was no way to predict which T-dnRAR mice would clear the infection. The livers and spleens of T-dnRAR mice at d10 post-infection had significantly higher *C. rodentium* burdens than WT littermates (Figures 5C,D). At d37 post-infection T-dnRAR mice had normal solid stools and histopathology scores that matched those of WT littermates that had cleared the infection (data not shown). To determine if RA treatment late in infection could induce clearance, 3 chronically infected T-dnRAR mice were treated with RA for 2 weeks starting at d31 post-infection (Figure 5E). The RA treatments failed to eliminate *C. rodentium* in the chronically infected T-dnRAR mice and the fecal shedding levels remained high (log 4–6 CFUs) out to d48 post-infection (Figure 5E). Initiating RA dosing at d7 post-infection also failed to induce clearance of the *C. rodentium* in all the T-dnRAR mice and 58% of the RA + T-dnRAR were chronically infected (Figure 5F). Mice lacking retinoid responsive T cells became asymptomatic reservoirs of C. *rodentium* infection that were refractory to RA treatment.

### T-dnRAR Mice Have Fewer T Cells but Robust Colonic IL-17 Following Infection

**Figure 6:**
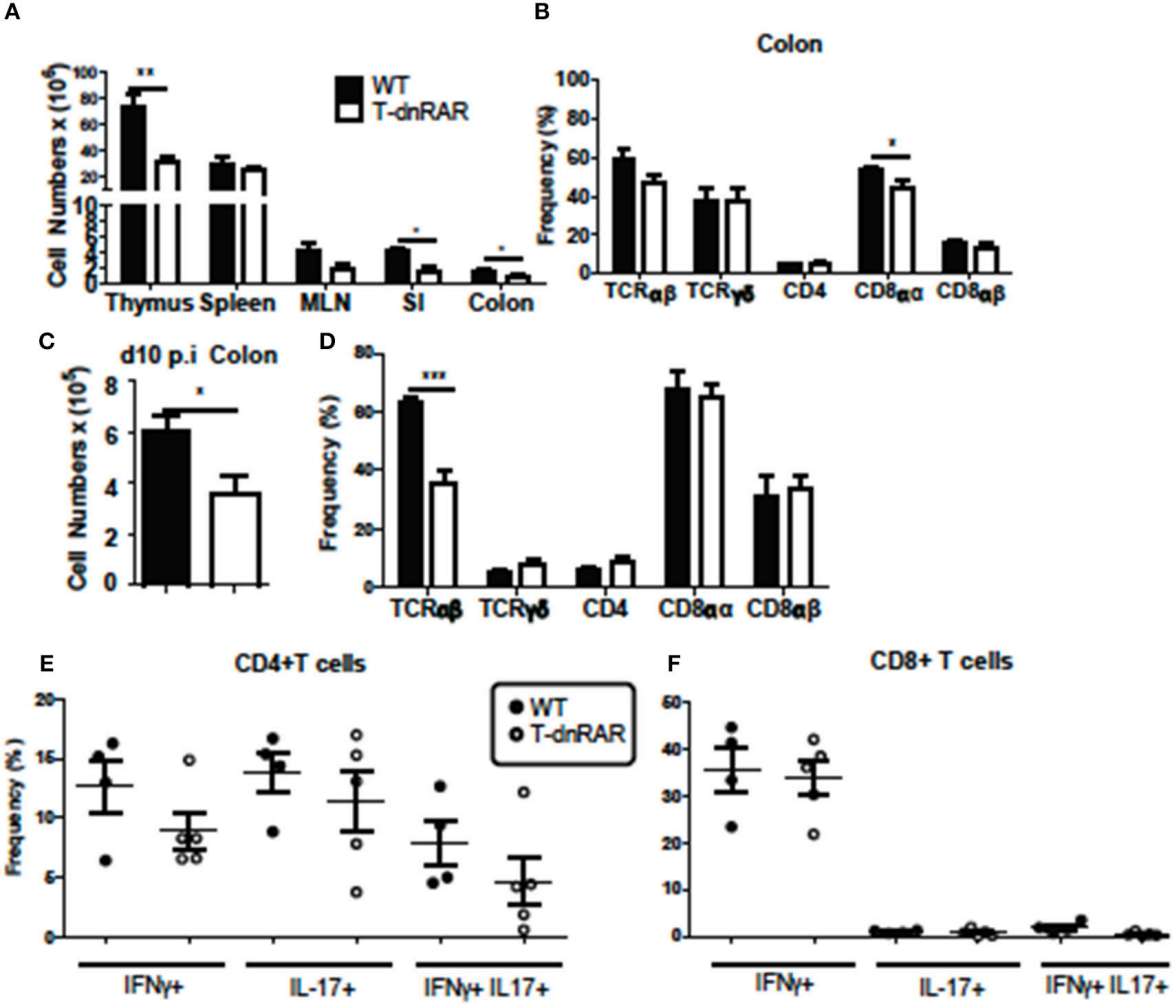
T-dnRAR mice have fewer T cells but robust IL-17 production in the colon. T cell populations in uninfected (*n* = 3–10/ group) and d10 infected (*n* = 4–5mice/group) T-dnRAR (T-dn) and WT littermates. **(A)** Total cell counts in thymus, spleen, MLN, SI, and colon of uninfected mice. **(B)** T cell frequencies in the colon of WT and T-dnRAR mice. **(C)** Total cell counts and **(D)** T cell frequencies in the colon at d10 post-infection. IFN-γ, IL-17 and double IFN- γ/IL17 producing **(E)** CD4+, and **(F)** CD8+ T cells in the d10 infected colon of T-dnRAR and WT littermates. Values are the means ± SEM of one **(C–F)** or two independent experiments **(A,B)**. Unpaired student's *T*-test, ^*^P < 0.05, ^**^P < 0.01, ^***^P < 0.0001.

Thymocytes and SI IEL were counted to determine if the number of T cells were different between T-dnRAR mice and WT littermates. There were fewer cells in the thymus, SI, and colon of T-dnRAR mice than in WT littermates (Figure 6A). The total cell numbers were the same in the spleen and mesenteric lymph nodes (MLN) of WT and T-dnRAR mice (Figure 6A). CD4+/CD8+ double positive (DP) thymocytes are the most frequent cell type in the thymus and 83% of the cells in the WT thymus were DP (Supplementary Figure 3A). By comparison, T-dnRAR mice had lower frequencies of DP thymocytes (67%) in the thymus as compared to their WT littermates (Supplementary Figure 3A). The decrease in the DP thymic population in T-dnRAR mice was reported previously (27). The spleen and MLN of T-dnRAR mice had higher CD4 frequencies and lower CD8 frequencies compared to WT littermates (Supplementary Figures 3B,C). No change was observed in TCRγδ+, CD8αα+, CD8αβ+, and CD4+ T cell frequencies in the colons of un-infected T-dnRAR mice (Figure 6B). The frequency of TCRαβ+ cells was lower in T-dnRAR spleens (Supplementary Figure 3C). The frequencies of CCR9+ CD4+ and CD8+ T cells in the spleens of T-dnRAR mice were significantly less than in WT littermates (Supplementary Figure 3D). Reduced CCR9 expression on splenic T cells in T-dnRAR mice was associated with significantly fewer cells in the SI and colon (Figure 6A). Infected T-dnRAR mice also had reduced total cell numbers in the colon compared to WT littermates (Figure 6C). In addition, the frequencies of TCRαβ+ T cells were lower in T-dnRAR colons than in WT colons (Figure 6D). However, colonic IFN-γ+, IL-17+, and IFN-γ/IL-17 double positive CD4+ and CD8+ T cell frequencies were the same in d10 infected T-dnRAR and WT mice (Figures 6E,F). T-dnRAR mice became chronically infected following *C. rodentium* infection, had fewer intestinal TCRαβ+ T cells, but similar frequencies of IL-17 and IFN-γ producing colonic T cells compared to WT littermates.

### CD11b+ Cells Produce IL-17 in the RA Treated A– Host

**Figure 7:**
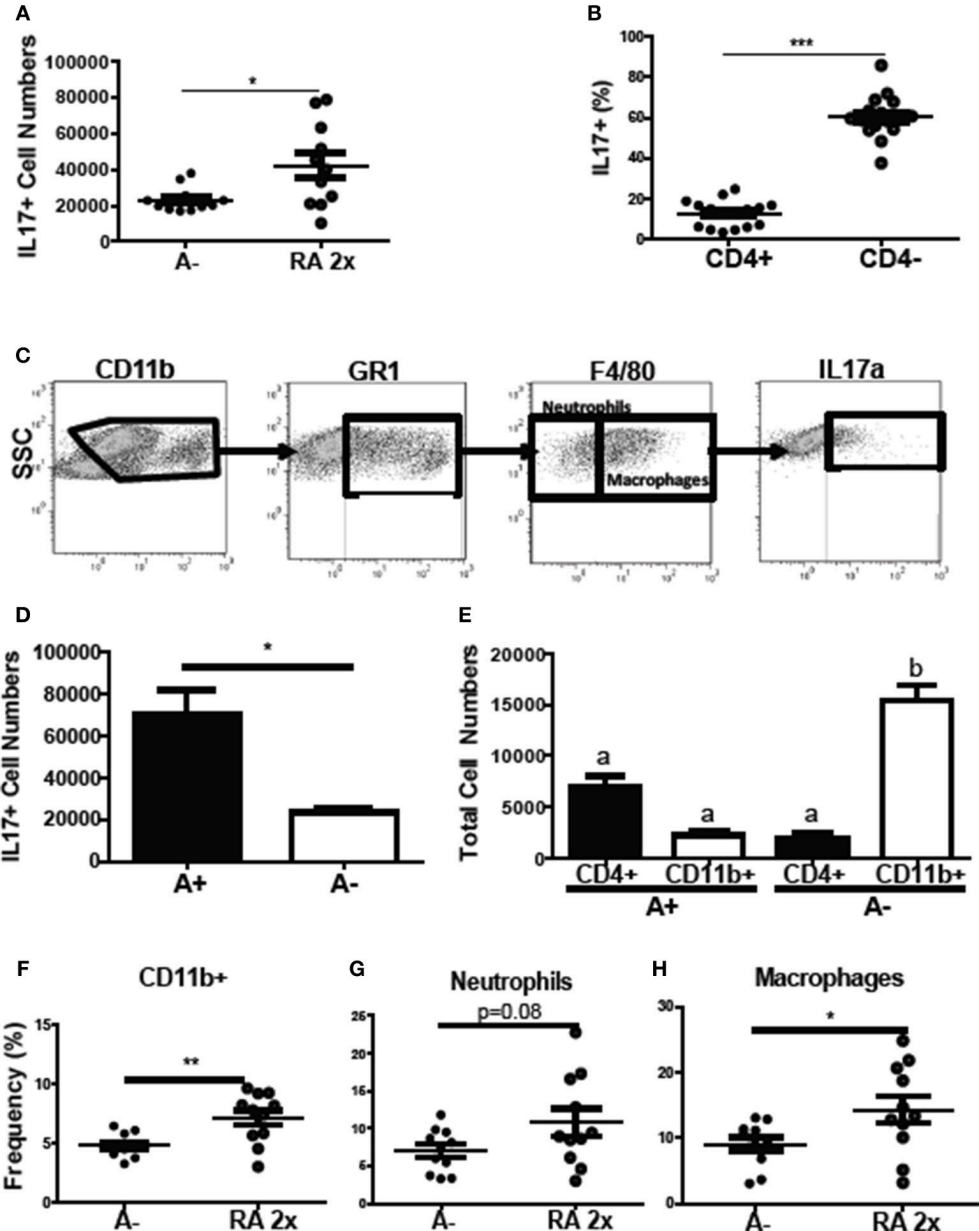
RA induced innate CD11b+ mediated IL-17 production in the colon of A– mice. The **(A)** number of IL-17+ cells and **(B)** CD4+ and CD4- cell IL-17+ frequencies in A– and A– 2X RA colon lamina propria. **(C)** Flow gating strategy for CD11b+ cells including neutrophils and macrophages. The numbers of IL-17+ cells in **(D)** A+ and A– colons and **(E)** in CD4+ vs. CD11b+ cells. The frequencies of IL-17 producing **(F)** CD11b+, **(G)** neutrophil (CD11b+/F480–), and **(H)** macrophage (CD11b+/F480+) cells. Values are means ± SEM of two combined experiments and *n* = 4–11/ group. Unpaired student's *T*-test, unpaired student's *T-*test with Welch's correction **(A,D,F–H)** ^*^P < 0.05, ^**^P < 0.01, ^***^P < 0.001. Kruskal-Wallis one-way ANOVA **(E)**. Values with different letters are significantly different from each other (*P* < 0.05).

To determine the source of the increased *il17* mRNA expression with RA treatment (Figure 4D), intracellular staining was done of the colon cells. The frequency of colonic IL-17 secreting cells increased following RA 2x treatments in *C. rodentium* infected A– mice (Figure 7A). The colonic IL-17 in A– and A– RA 2x treated mice was primarily produced by a CD4 negative population and not by CD4+ Th17 cells (Figures 7B,C). The total number of IL-17 producing cells was higher in the A+ infected colon compared to the A– infected colon (Figure 7D). CD4+ T cells were the predominant producers of colonic IL-17 in the A+ host (Figure 7E). In A– mice, CD4+ T cells accounted for only 12% of the colonic IL-17 producing cells (Figure 7B). The IL-17 mean fluorescence intensities were not different in either the CD11b+ or T cells in the A+ or A– hosts (data not shown). RA treated A– mice had significantly elevated frequencies of IL17 secreting CD11b+ cells compared to untreated A– mice (Figure 7F). The RA treatments had no effect on the numbers of CD11b+, CD11b+/F480– (neutrophils) or CD11b+/F480+ (macrophage) cells in the colon (Supplementary Figure 4). The frequency of neutrophils (*P* = 0.08) and macrophages (*P* < 0.05) that produced IL-17 increased in the RA 2x treated mice compared to the A– controls (Figures 7G,H). There was no effect of RA 2x treatment on IL-17 production in the colons of infected A+ T-dnRAR mice (Supplementary Figure 3E). RA 2x treatment of A– mice induced CD11b+ innate cells to produce IL-17.

### Retinoid Signaling in LysM Expressing Cells Is Required for Clearance of *C. rodentium*

**Figure 8:**
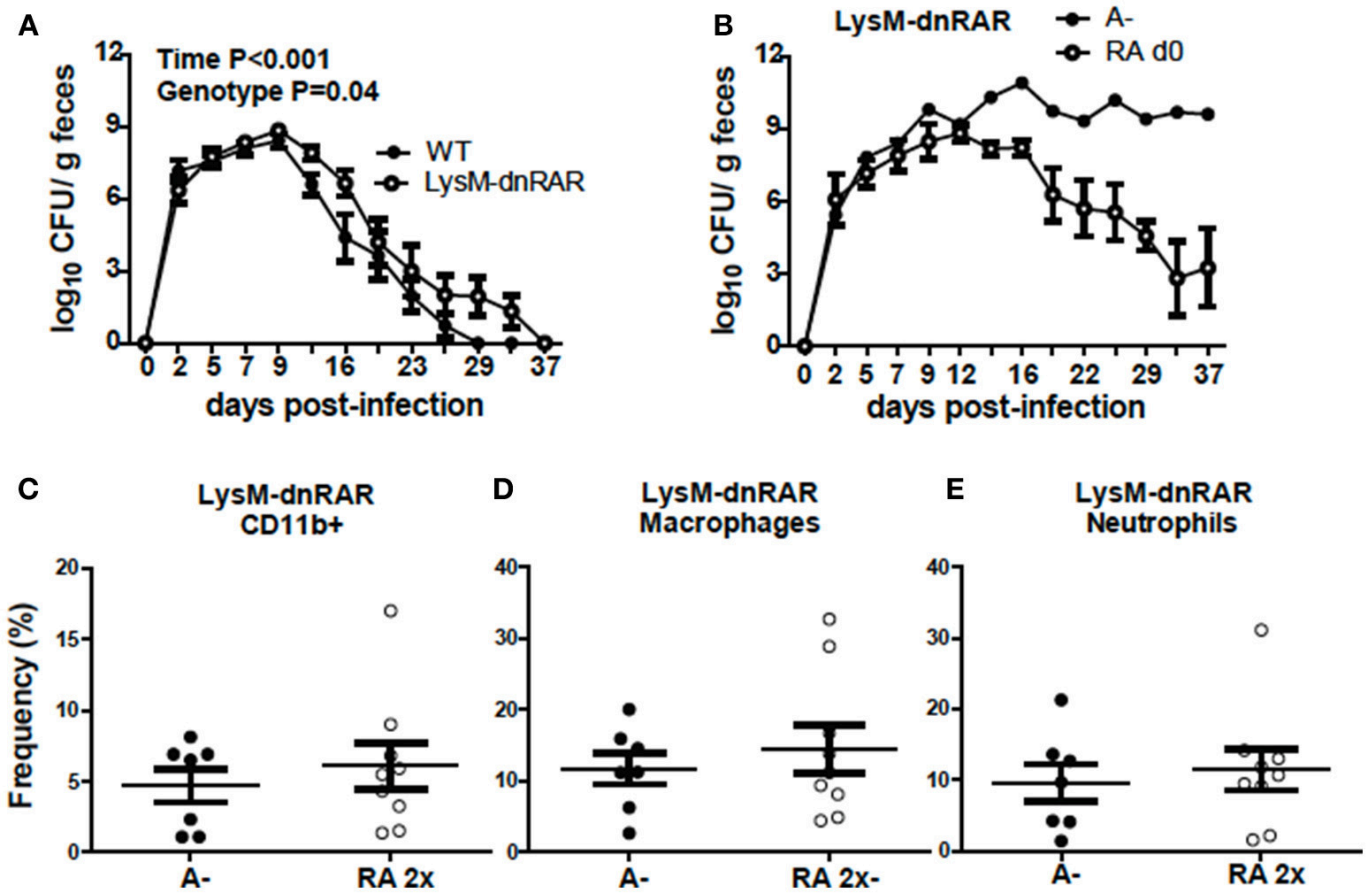
Retinoid signaling in LysM expressing cells is required for clearance of *C. rodentium*. *C. rodentium* fecal shedding kinetics in **(A)** A+ WT and A+ LysM-dnRAR mice (*n* = 9/ group) and **(B)** LysM-dnRAR A– and A– RA d0 mice (*n* = 1–3/ group). IL17 secreting **(C)** CD11b+ cell **(D)** neutrophil, and **(E)** macrophage frequencies were quantified in the colons of infected control and RA 2x treated A– LysM-dnRAR mice (*n* = 7–9/ group). Values are the means ± SEM of two to three independent experiments and *n* = 3–7 mice per group. Two-way ANOVA with Bonferroni post-test **(A)** and unpaired student's *t-*test **(C–E)**. There were not enough surviving A– LysM-dnRAR mice perform statistical analyses for **(B)**.

To determine if RA directly regulates CD11b+ cell function, LysM-dnRAR mice were generated and infected with *C. rodentium*. The *C. rodentium* shedding kinetics of the A+ WT (fl/fl) littermates resembled those in Figure 1 and the shedding kinetics of A+ WT mice reported previously (4). The A+ WT littermates cleared *C. rodentium* by d29 post-infection (Figure 8A). A+ WT and A+ LysM-dnRAR mice all survived *C. rodentium* infection; however, A+ LysM-dnRAR mice took significantly longer to clear the infection than the WT littermates (Figure 8A). A– LysM-dnRAR mice developed a lethal infection in 50% of the mice with the remaining A– LysM-dnRAR mice shedding high levels of *C. rodentium* in the feces (Figure 8B). Treatment of A– LysM-dnRAR mice at d0 with RA prevented lethality, but surviving mice became chronically infected with *C. rodentium* (Figure 8B). Two RA doses at d7 post-infection in LysM-dnRAR mice had no effect on the frequencies of IL-17 secreting CD11b+ cells, neutrophils, or macrophages in the colon of the mice (Figures 8C–E). RA treatment failed to induce IL17 production by CD11b+ cells in LysM-dnRAR mice. LysM expressing CD11b+ cells in the colon are critical retinoid targets required for effective elimination of *C. rodentium* infection.

## Discussion

RA treatment of infected A– mice induced colonic IL17 production by CD11b+ cells, which was associated with the clearance of *C. rodentium* infection. Mice that expressed the dnRAR in either T cells or CD11b+/LysM expressing cells were unable to clear *C. rodentium* with the same kinetics as WT. There was no effect of RA on IL-17 production in the colon of mice that expressed the LysM-dnRAR. A robust IL-17 response is critical for the clearance of several mucosal pathogens including *C. rodentium* (8, 28–30). In A+ mice, T cells provided most of the IL-17 in the infected colon. It is well-established that RA inhibits Th17 differentiation and function *in vitro*; RA treatment reduced T cell derived IL-17 and improved disease outcomes in several intestinal inflammation models that used A+ animals (14–16). A– mice have fewer intestinal lymphocytes likely due to reduced α4β7 and CCR9 expression and impaired intestinal homing (4, 31–34). RA treatments did not restore the A– immune cells and metabolic profiles to that of an A+ mouse. Very little has been published on leukocyte mediated production of IL-17 or the effect(s) RA may have on innate cells producing IL-17. In CD4 T cells, RA inhibited the expression of the receptors for IL-6 and IL-23 that are critical in the differentiation of Th17 cells (35, 36). A population of RORγt+ IL-17 producing neutrophils have been identified and their function was determined to be RORγt and IL-6 dependent (35). The enhanced colonic IL-17 production in RA treated A– mice could be due to (1) RA regulating IL-6 and IL-23 signaling indirectly that then affects IL-17 in myeloid cells or (2) direct RA regulation of IL-17 production in myeloid cells. The induction of IL-17 production by RA in CD11b+ cells was associated with the ability of the A– mice to clear *C. rodentium* infection.

A conclusion of the present study is that retinoid signaling in T cells is required for clearance of *C. rodentium*. Over 60% of T-dnRAR mice were unable to clear the infection and RA treatment failed to induce clearance in chronically infected T-dnRAR mice. It is unclear why 40% of the T-dnRAR mice cleared the infection with the same kinetics as the WT mice. However, both WT and T-dnRAR mice had robust production of IL-17 and IFN-γ in the gastrointestinal tract suggesting that the cytokine response meets an immunological threshold for some, but not all mice. Others have shown that T-dnRAR mice have more Th17 cells than WT mice and that antigen specific Th17 cells accumulate in the T-dnRAR gastrointestinal tract and periphery (37). The T-dnRAR Th17 cells develop as a result of failed Th1 development (37). The inability of the T-dnRAR mice to clear *C. rodentium* could be because of failed Th1 development. None of the T-dnRAR mice developed a severe and lethal infection, which supports a role for retinoids signaling in T cells for clearance but not prevention of lethality following *C. rodentium* infection. The results of the current study are confounded by the role of vitamin A in development and gastrointestinal homing of T cells. Regardless, retinoid signaling in T cells is required for efficient clearance of *C. rodentium*.

A– WT, A– LysM dnRAR, and T-dnRAR mice all become chronically infected with *C. rodentium*. There were no outward symptoms of diarrhea or weight loss in the chronically infected mice and histopathology of the colon showed resolution of inflammation. *C. rodentium* infection of germfree mice resulted in diarrhea and inflammation in the gastrointestinal tract which resolved after several weeks, even though the mice still had high numbers of *C. rodentium* being shed in the feces (38). Late RA treatment of chronically infected A– mice is no longer effective for clearance of *C. rodentium*. If *C. rodentium* infection is not cleared, inflammation and symptoms resolve, but the mice become chronic asymptomatic carriers that are refractory to retinoid interventions.

Millions of children in resource limited countries are vitamin A deficient and at increased risk of developing more severe enteric infections due to their vitamin A status (1, 2). The data presented in this study suggest that RA and retinoid receptor signaling are critical for host protection from enteric infections. RA treatment of A– mice did not restore mucosal immune responses and/or the metabolic phenotype of the mice. Contrary to what has been published in A+ animals (14–16), RA treatment *in vivo* induced colonic IL-17 production in *C. rodentium* infected A– mice. In addition, the data suggest that strategies that replete the previously vitamin A deficient host with retinoids may not completely recover the metabolic and immunological effects of the vitamin A sufficient state. Although it is true that humans in developing countries are not treated with RA, but instead are administered retinol or retinyl palmitate to replete vitamin A stores, the data does suggest that, there could be long-term metabolic and immunologic consequences to developmental vitamin A deficiency. The data also suggest that RA treatments could be useful for treating A– individuals with acute enteric infections and minimizing the number of asymptomatic carriers, thereby preventing the spread of infection within susceptible populations.

## Ethics Statement

This study was carried out in accordance with regulations and recommendations of the Institutional Animal Care and Use Committee (IACUC) of The Pennsylvania State University, University Park, PA, USA. The protocol was approved by the IACUC committee of The Pennsylvania State University, University Park, PA, USA.

## Author Contributions

MC, LS, KM, and YT conceptualized and designed the experimental studies. LS, KM, and YT performed the experiments and acquired, and analyzed the data. C-HW quantified serum retinol levels and MK scored histology slides. LS drafted the manuscript with the help of MC, YT, and C-HW. MK, AP, AR, and MC critically revised the manuscript. All authors approved the publication of the manuscript.

### Conflict of Interest Statement

The authors declare that the research was conducted in the absence of any commercial or financial relationships that could be construed as a potential conflict of interest.
