# Supplementary material for: Retinoic Acid Mediated Clearance of Citrobacter rodentium in Vitamin A Deficient Mice Requires CD11b+ and T Cells
Source: Front Immunol. 2019 Jan 8;9:3090. doi: 10.3389/fimmu.2018.03090 (PMC6331472; doi:10.3389/fimmu.2018.03090)
Supplement: Supplementary file 1 [file Data_Sheet_1.pdf]

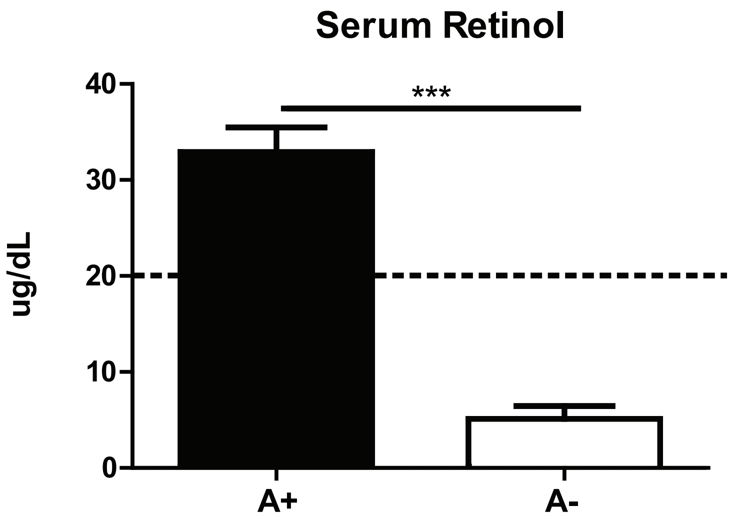

Supplemental Figure 1: Serum retinol values in 7-12 wk old A+ and A- mice Values are from pooled samples and several different experiments (n=8-18/group). Dotted line indicates deficient/sufficient cutoff (20 ug/dL) Unpaired student's T test with Welch's correction. ***P<0.0001.

Supplementary Table 1: RT-PCR primer sequences

HPRT:	F: 3’-GTAATGTTATCGAGAAGTCAGAC-5’<br>R: 3’-CACCTTCTATATTAACTGTGACC-5’
GAPDH:	F: 3’-TAGCTTCCACCTTCTACACCAAA-5’<br>R: 3’-GATGTCGTTGTCCCACCACC-5’
FOXP3:	F: 3’-GACGTATCGAGGGTCGAAGA-5’<br>R: 3’-CGAGAACGACGTAGCATCG-5’
RORC:	F: 3’-AGGAGGGCACTTTTCTCCA-5’<br>R: 3’-GTTACACCCTCTACACCCT-5’
IL-17A:	F: 3’-TGTGTCTACTTCGAGAGGGAC-5’<br>R: 3’-TAGTAGGGAGTTTCGACTCG-5’
IL-22:	F: 3’-AGACCTACAAGACCAGCAGT-5’<br>R: 3’-CACCTCTCTAGTTCCGCTAA-5’
IL-6:	F: 3’-AGGACTAATATAGGTCAAACCCATCG-5’<br>R: 3’-AAGACCTCATGGTATCGATGGACC-5’
REGIIIy	F: 3’-AAAACTAGTACCTCCTGTCCTT-5’<br>R: 3’-ACTTGGGTTGTCTCCACCTAC-5’

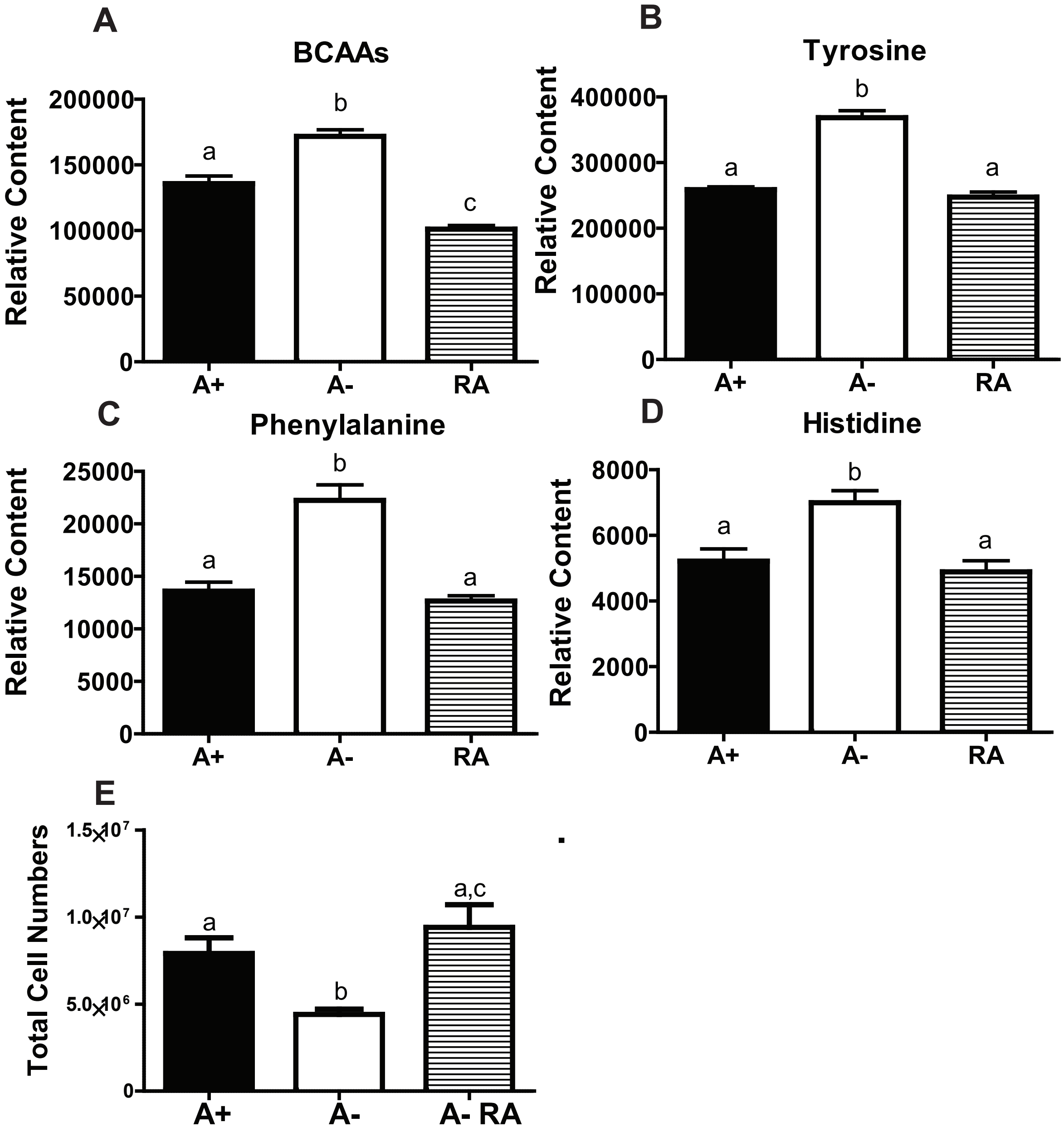

Supplementary Figure 2: Liver amino acid metabolites and intestinal cell counts in A+, A-, and A- RA mice. A) BCAAs, B) tyrosine, C) phenylalanine, and D) histidine levels were measured by proton NMR (n=6). E) SI IEL cell counts were quantified for each treatment group (n=8-9). Values are the means ± SEM of two to three independent experiments One way ANOVA with Bonferroni post-test, groups with different letters are significantly different from each other, P<0.05.

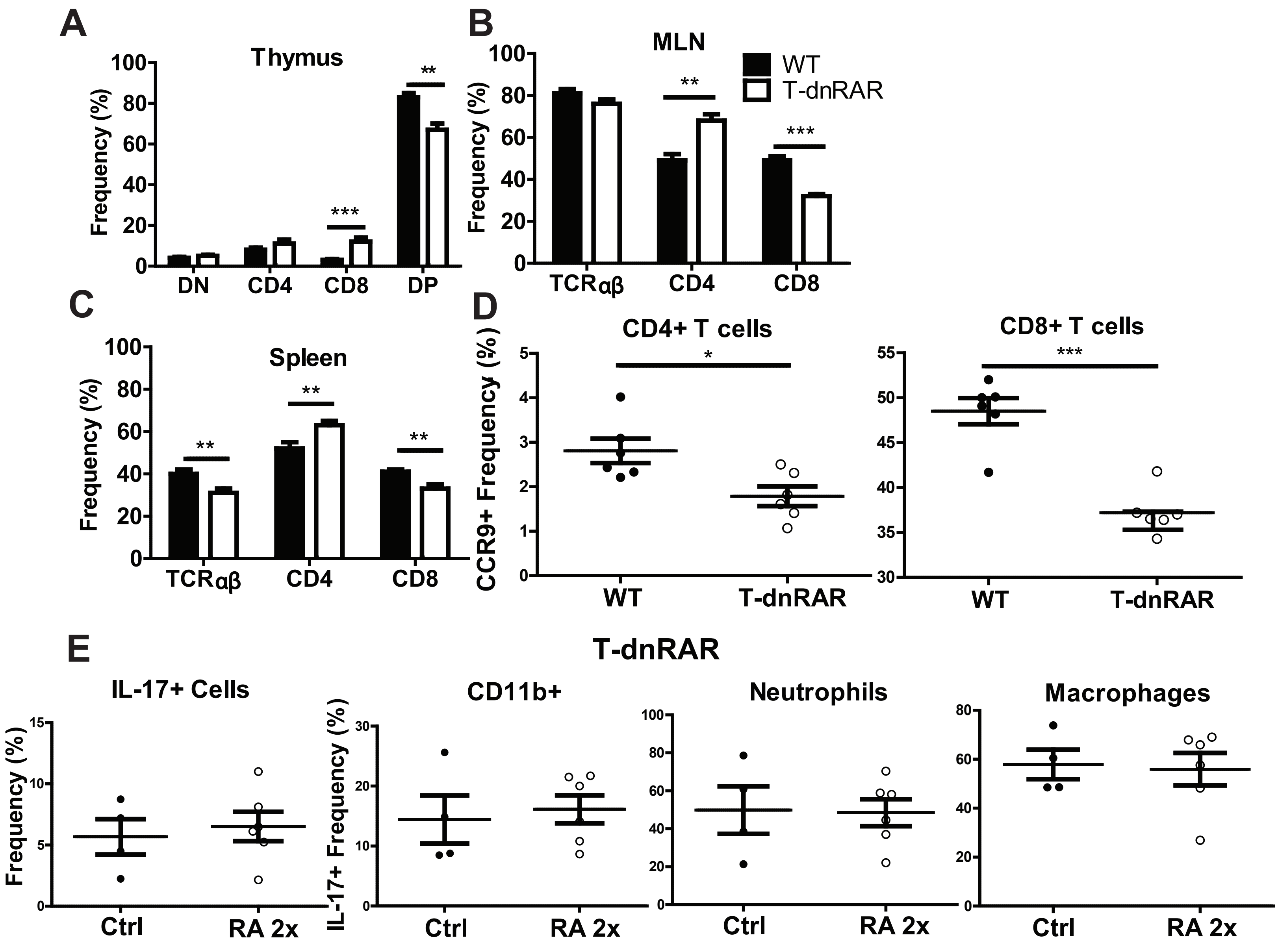

Supplemental Figure 3: T cell populations and RA effects on IL-17. T cell populations were quantified in uninfected T-dnRAR and WT littermates (n=3-10/ group). T cell frequencies in the A) thymus, B) mesenteric lymph node (MLN) and C) spleen. D) CCR9 expression in spleen CD4+ and CD8+ T cells. E) IL17 producing populations in the colon lamina propria of d10 infected T-dnRAR and RA 2x treated T-dnRAR mice in colon, CD11b+, neutrophils and macrophage (n=4-6/ group). Values are means ± SEM of two independent experiments (A-C). Unpaired student's T test *P<0.05, **P<0.01, ***P<0.001.

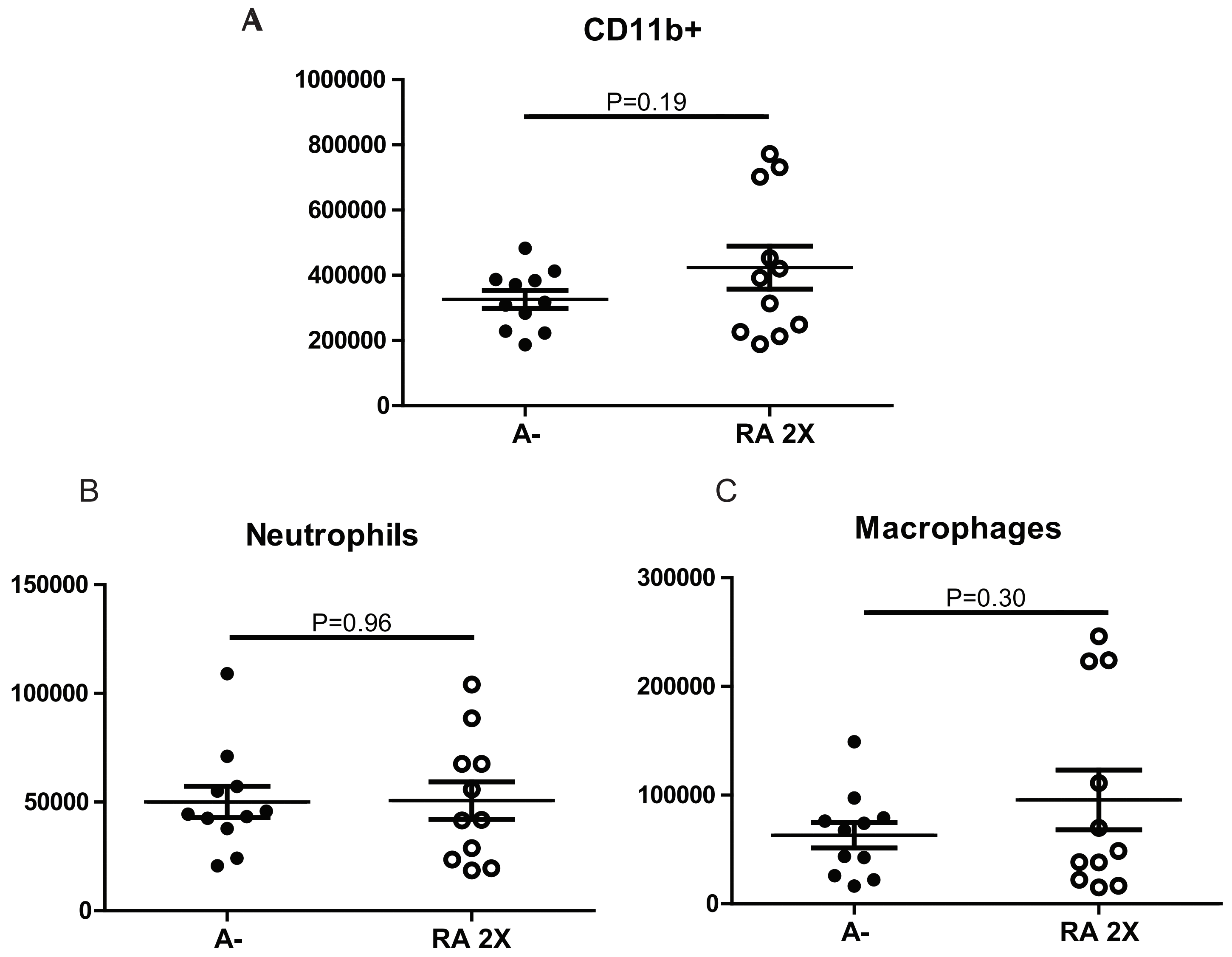

Supplemental Figure 4: RA treatment did not affect colonic neutrophil or macrophage infiltration in infected A- mice. Colon LP A) CD11b+, B) neutrophil (CD11b+GR1+F4/80-), and C) macrophage (CD11b+GR1+F4/80) cell numbers. Values are means ± SEM of two combined experiments and n=11/ group. Unpaired student's T test (B) or unpaired student's T test with Welch's correction (A, C).
